# Identification of environmental and genetic factors that influence warfarin time in therapeutic range

**DOI:** 10.1590/1678-4685-GMB-2019-0025

**Published:** 2020-02-10

**Authors:** Mariana R. Botton, Patrícia P. Viola, Mariana R. Meireles, Estela M. Bruxel, Priccila Zuchinali, Eliane Bandinelli, Luis E. Rohde, Tiago L. L. Leiria, Joyce Y. Y. Salamoni, Arthur P. Garbin, Mara H. Hutz

**Affiliations:** 1 Universidade Federal do Rio Grande do Sul, Departamento de Genética, Porto Alegre, RS, Brazil.; 2 Hospital de Clínicas de Porto Alegre, Porto Alegre, RS, Brazil.; 3 Fundação Universitária de Cardiologia, Instituto de Cardiologia do Rio Grande do Sul, Porto Alegre, RS, Brazil.

**Keywords:** CYP2C9, VKORC1, ASPH, TTR, warfarin

## Abstract

Warfarin is an oral anticoagulant prescribed to prevent and treat thromboembolic disorders. It has a narrow therapeutic window and must have its effect controlled. Prothrombin test, expressed in INR value, is used for dose management. Time in therapeutic range (TTR) is an important outcome of quality control of anticoagulation therapy and is influenced by several factors. The aim of this study was to identify genetic, demographic, and clinical factors that can potentially influence TTR. In total,422 patients using warfarin were investigated. Glibenclamide co-medication and presence of CYP2C9*2 and/or *3 alleles were associated with higher TTR, while amiodarone, acetaminophen and verapamil co-medication were associated with lower TTR. Our data suggest that TTR is influenced by co-medication and genetic factors. Thus, individuals in use of glibenclamide may need a more careful monitoring and genetic testing (CYP2C9*2 and/or *3 alleles) may improve the anticoagulation management. In addition, in order to reach and maintain the INR in the target for a longer period, it is better to discuss dose adjustment in office instead of by telephone assessment. Other studies are needed to confirm these results and to find more variables that could contribute to this important parameter.

## Introduction

Oral anticoagulants are extensively used in clinical practice to prevent and treat thromboembolic disorders (Hylek, 2013). Coumarins are the most used class of oral anticoagulants. Among the coumarins, warfarin is the main oral anticoagulant prescribed worldwide. It is a vitamin K antagonist (VKA) that competitively inhibits the vitamin K epoxide reductase complex 1 (VKORC1), which is an essential enzyme for the coagulation cascade. Vitamin K is an important cofactor to activate the procoagulant factors II, VII, IX, and X. Its effect is measured by the International Normalized Ratio (INR) value using the prothrombin test. Most patients have to maintain a range between 2.0 and 3.0 INR (depending on indication, the target INR can be higher). Warfarin has a narrow therapeutic window, thus, its effect must be strictly controlled (Ageno *et al.*, 2012).

Poor coagulation control is associated with greater risk of bleeding and thromboembolic events (Connolly *et al.*, 2008). Several studies proposed different pharmacogenetic algorithms (see for instance Botton *et al.*, 2011) to determine the individual dose for each patient, but until now, most of them explained up to a maximum of 60% of warfarin dose variation. The time in therapeutic range (TTR) is another important outcome to check for INR control quality. Bleeding or thrombosis events can be serious problems. Physicians usually start treatment with lower doses to avoid bleeding and then, according to INR results, they adjust the dose until INR reaches the therapeutic target. However, patients who need higher doses generally stay for longer time with INR out of target, being on thrombosis risk.

This can be prevented by avoiding warfarin over- or under-dosing. Moreover, the lowest percentage of time that patient spend in target INR is associated with bleeding, stroke and/or mortality (Connolly *et al.*, 2008). Several factors can influence TTR, among them, therapy duration, the site where anticoagulation is monitored, type of management, age, non-adherence to therapy, gender, ethnicity, polypharmacy, psychiatric disorders and other diseases, genetic factors, time for repeating the test after an out-of-range INR, and selection of target INR (Macedo *et al.*, 2015). However, the literature is very controversial (Apostolakis *et al.*, 2013 Pokorney *et al.*, 2015). Among the genetic factors, polymorphisms in the *CYP2C9* and *VKORC1* genes have already been associated with TTR (Park *et al.*, 2015, 2017; Wypasek *et al.*, 2015; Bryk *et al.*, 2018). More recently, a GWAS reported that two SNPs (rs17791091 and rs4379440) in *ASPH* gene are associated with TTR (Eriksson *et al.*, 2016). As far as we know, up to the present day, there is no study that tried to replicate this GWAS result.

In order to guarantee a safe and effective treatment with warfarin, the knowledge of factors related to TTR are important in addition to algorithms for drug adjustment. Therefore, the aim of this study was to investigate the association between genetic (*CYP2C9*, *VKORC1*, and *ASPH*) and environmental factors associated with TTR.

## Materials and Methods

### Subjects

A total of 422 patients were recruited at two university hospitals, Instituto de Cardiologia – Fundação Universitária de Cardiologia and Hospital de Clínicas de Porto Alegre. Both hospitals have different types of management, but both involve clinical *gestalt*. One of them follows up treatment basically by phone. Patients go to the hospital, have their blood collected for the INR test and then, a health provider calls them to guide dose adjustment, if needed. The other hospital follows up the anticoagulation treatment by office appointment. The patients go there, have their blood collected for INR test, and then wait about 1 hour to have the result and talk to a health provider to discuss the therapy. We monitored the overall compliance by making questions to the patients as to how warfarin was used and other habits related to therapy. Besides that, the INR history also helped to identify if a patient has stopped or not the use of medication.

The patients included in this investigation were in anticoagulation therapy for at least 24 months. The inclusion criteria were: use of warfarin for at least 24 months after the achievement of target INR, and an age of ≥18 years. Exclusion criteria were: useof warfarin for less than 24 months, and being younger than 18 years. Patients had their INR tested monthly.

To evaluate TTR, the last 24 months of anticoagulation were considered. INR values of patients who had to suspend the anticoagulation for a period were not considered. TTR was calculated based on the Rosendaal linear interpolation (Rosendaal *et al.*, 1993). The 24 month cut-off was chosen to avoid any kind of bias due to shorter observation time.

All patients completed a questionnaire about their demographic and clinical data and received information about the study. All drugs used by patients in the period of investigation were considered, but due to sample size, not all were statistically analyzed. This study was approved by the Ethics Committees of the Instituto de Cardiologia – Fundação Universitária de Cardiologia and of the Hospital de Clínicas de Porto Alegre. All subjects provided written informed consent.

### DNA extraction and genotyping

Genetic material was extracted from whole blood using the PureLink Genomic DNA Purification Kit(Invitrogen, Carlsbad, CA, USA) according to the manufacturer’s instructions. Prothrombin time was measured at the hospitals Central Laboratories. CYP2C9*2, CYP2C9*3,VKORC1rs9923231, and *ASPH*rs17791091, as well as rs4379440SNPs were genotyped using TaqMan SNP Genotyping Assays (Thermo Fisher Scientific, Carlsbad, CA, USA) according to the manufacturer’s recommended protocols.

### Statistical analysis

Hardy-Weinberg Equilibrium was estimated by the chi-square test. Linear regression was used to estimate the influence of genetic and non-genetic factors on TTR through the enter method. Variables with *p*>0.05 were removed one at a time, always choosing the variable with higher *p*-value. ANOVA or Student’s *t-*tests were used to select variables for inclusion in the regression model. Variables with *p*<0.10 were included in the linear regression, but only variables with *p*<0.05 in the regression were kept in the model. All analyses were performed with the SPSS 18.0 software.

## Results

The patients’ average age was 62.3 ± 13.9 years. Most patients were males (53.7%) of European ancestry (93.6%). Mean TTR was 59.5% of treatment days. The average warfarin dose was 33.0 mg/week (Table 1). The indications for anticoagulation are also shown in this table. Table 2 shows the most frequent comorbidities and the most used medicines by the patients from this sample.

**Table 1:** Clinical and demographic characteristics of patients according to TTR.

	TTR ≥ 65% (n = 174)	TTR < 65% (n = 248)
Weekly warfarin dose	33.3 ± 13.6 mg	32.7 ± 15.9 mg
Age	62.6 ± 13.6	62.2 ± 14.2
Body weight	76.3 ± 12.9 kg	73.5 ± 14.0 kg
Height	1.67 ± 0.1 m	1.65 ± 0.1 m
BMI	27.4 ± 4.1	27.1 ± 4.5
Atrial fibrillation	56.2%	53.9%
Aortic prosthesis	18.1%	24.1%
Mitral prosthesis	26.2%	27.0%
Gender (male)	57.5%	51%
Ethnicity (European ancestry)	89.7%	90.3%

**Table 2:** Comorbidities and co-medications of patients included in the investigated sample.

Comorbities*	n
Cardiopathy	110
Dislipidemy	124
Diabetes	88
Hypertension	280
Hypothyroidism	27
Renal failure	31
**Other medications** *	**n**
AAS	156
Amiodarone	36
Anlodipine	48
Atenolol	56
Captopril	117
Carbamazepine	9
Digoxin	98
Enalapril	138
Spironolactone	40
Furosemide	158
Glibenclamide	22
Hydralazine	27
Hydrochlorothiazide	121
Isosorbide	35
Levothyroxine	30
Losartan	33
Metformin	54
Metoprolol	162
Omeprazole	87
Propranolol	45
Simvastatin	178

* The subjects can have more than one comorbity, or be in use of more than one co-medication.

A significant difference in TTR between the two different types of management was observed. The group of patients followed by an office appointment had higher TTR than patients followed by phone (*p*<0.001). Therefore, “type of management” was included in the regression model as a confounder (Table 3). Glibenclamide, verapamil, acetaminophen and amiodarone use and *CYP2C9* gene were associated with TTR. Comorbidities such as diabetes and hypertension were not associated with TTR. Patients who use glibenclamide and/or have a CYP2C9*2 and/or *3 allele stayed more time in therapeutic range. On the other hand, the use of amiodarone, verapamil, and/or acetaminophen as co-medication was associated with lower TTR. Variables with higher influence were glibenclamide, amiodarone, and the *CYP2C9* gene, in this sequence. Other studied factors, including *VKORC1* and *ASPH* variants, were not associated with TTR.

**Table 3:** Final regression model for TTR.

Variable	B coefficient	Beta coefficient	*p*-value	Partial r^2^
CYP2C9 *2 and/or *3	5.255	0.130	0.006	0.017
Amiodarone	-9.029	-0.133	0.005	0.018
Glibenclamide	13.385	0.157	0.001	0.024
Acetaminophen	-16.097	-0.108	0.023	0.011
Verapamil	-11.388	-0.096	0.045	0.009
Type of management^a^	-7.639	-0.192	<0.001	0.036

a The regression was made using “type of management” as a confounder variable.

## Discussion

In the present study, patients followed by an office appointment had higher TTR than those followed by telephone calls. Patients treated in office usually receive more attention and better guidance. Moreover, in office, the health provider can better assess possible changes in a patients habits. A previous study showed that patients monitored by telephone have higher levels of extreme out-of-range INR than patients monitored in office (Stoudenmire *et al.*, 2014), which corroborates with our findings. However, in both kinds of managements, the average TTRs were those expected for patients to receive benefits from being in anticoagulation therapy(Connolly *et al.*, 2008).

The anticoagulation control is considered effective if patients stay stable about 65% of the time (Connolly *et al.*, 2008). Moreover, individuals with TTR lower than 60% have higher rates of mortality and major bleeding (White *et al.*, 2007). The benefits of anticoagulant therapy are larger than that with dual antiplatelet therapy in prevention of stroke in atrial fibrillation only if patients achieve a 58% - 65% TTR threshold (Connolly *et al.*, 2008). In the present study, the average TTR was about 60%, which is the TTR achieved in most of the randomized controlled trials comparing warfarin and new oral anticoagulants(Connolly *et al.*, 2008). Socioeconomic status is related to adherence to treatment, which can influence the quality of anticoagulant therapy and, consequently, decreases TTR. In the present study, socioeconomic status was not analyzed. However, all patients were recruited from two public hospitals; therefore, most of them have low socioeconomic status. In addition, patients who clearly did not have a good adherence to treatment were excluded. TTR varies by centers and country between 54% and 73%, and adherence to warfarin treatment algorithms is associated with higher levels (Van Spall *et al.*, 2012). Some strategies that can be used to improve TTR may comprise self-testing for specific patients, more frequent tests, and caregiver designation for those patients with cognitive impairment. It is also recommended to make tests more often after hospital discharge (Hylek, 2013).

In the present study, patients who have one or more *CYP2C9* variants presented higher TTR. Tomita *et al.* (2013) reported that *CYP2C9* and *VKORC1* polymorphisms were related to warfarin dose, but were not TTR determinants. On the other hand, some studies showed that *VKORC1* polymorphisms, but not *CYP2C9*, are associated with lower time in therapeutic range (Park *et al.*, 2015, 2017; Bryk *et al.*, 2018). Also, *CYP2C9* variants were associated with lower TTR values in previous studies (Wypasek *et al.*, 2015, 2016), but our results did not corroborate this data since we found that *CYP2C9* variants are associated with higher TTR values. However, they analyzed only the first 3 months of follow up, while the present investigation analyzed 24 months.

The GWAS results reported by Eriksson *et al.* (2016) showed that the *ASPH* gene was associated with TTR. ASPH plays an important role in calcium homeostasis. Its longest isoforms are responsible by the aspartic acid or asparagine residues hydroxylation of protein C and coagulation factors VII, IX, and X (Eriksson *et al.*, 2016). However, the present study did not confirm this GWAS results. Nevertheless, some differences between the GWAS analysis and the present study are worthy of note. All patients recruited for the GWAS had the indication for anticoagulation related to atrial fibrillation, whereas the present investigation included patients with both atrial fibrillation and valve replacement as indications for anticoagulation. Moreover, Eriksson and colleagues (Eriksson *et al.*, 2016) included variables such as CHADS_2_ score, previous stroke, baseline INR, not investigated in the present study.

Gender, age, body weight, comorbidity and co-medications were reported to influence TTR (Macedo *et al.*, 2015). In the present investigation, only co-medications were kept in the regression model. Patients co-medicated with Glibenclamide spent more time in therapeutic range. This association was independent of diabetes or other anti-diabetic medications used by the patients. Glibenclamide is metabolized mainly by CYP2C9 and to a lesser extent by CYP3A4 enzymes, which can explain the connection found between warfarin and glibenclamide. In turn, amiodarone, verapamil, and acetaminophen decrease TTR. Amiodarone is a CYP2C9 inhibitor, while verapamil is metabolized by CYP2C9 and CYP3A4 enzymes.

Macedo *et al.* (2015) reported that the use of pain medication (acetaminophen, NSAIDs, or opioids) is associated with poor anticoagulation control in patients with atrial fibrillation and venous thromboembolism, which is in line with the present results. According to the Pharmacological Management of Persistent Pain in Older Persons guideline, acetaminophen is safer than others NSAIDs because it is not associated with significant gastrointestinal bleeding and does not influence platelet function. Some conditions that explain its use is osteoarthritis and low back pain, and it is recommended as first-line therapy for pain to elderly(American Geriatrics Society Panel on the Pharmacological Management of Persistent Pain in Older Persons, 2009). In this study, the median age of the groups is 62 years old, which can explain its use. Several studies have been published warning about an acetaminophen-warfarin interaction, showing that it increases INR and, therefore, can lead to being out of TTR. There is no recognized mechanism that explains this interaction, but Lopes *et al.* (2011) proposed a mechanism based on the pharmacodynamics of acetaminophen.

The model based on our sample explains only a small fraction of TTR variability (9.3%), which means that there are other important factors that were not addressed in this study. It is possible that factors that change during treatment might have a greater impact on TTR, like the number of times that patients have their prescription changed, changes in vitamin K consumption during treatment, comorbidities, and other medications. This could also explain the great discrepancy in results observed among the studies.

Besides the genetic factor, this study showed that variables of easy access, like co-medications, can influence INR control quality outcomes. Special attention and better management of these factors can improve warfarin therapy and, consequently, offer a therapy with higher efficacy and safety for the patients. Glibenclamide amiodarone, acetaminophen, and verapamil users that are also CYP2C9*2 and/or *3 allele carriers may need a more careful monitoring. Moreover, the present results about patient assessment may encourage hospitals to approach patients by in office appointments. Other studies are needed to confirm these results and to find more variables that could contribute to this important parameter.
